# Clinical impact of adverse reactions to medicines used in the treatment of ADHD

**DOI:** 10.1192/j.eurpsy.2026.10416

**Published:** 2026-07-31

**Authors:** M. Tomas Chicarino, G. Alves Andrade dos Santos

**Affiliations:** São Leopoldo Mandic, Araras/SP, Brazil

## Abstract

**Introduction:**

Attention deficit hyperactivity disorder (ADHD) is classified as a neurodevelopmental disorder characterized by a persistent pattern of inattention and/or hyperactivity and impulsivity. Epidemiological studies indicate a worldwide prevalence of between 5% and 7% in children, with persistence rates in adulthood estimated at between 2.5% and 3.5%. A therapeutic approach with pharmacotherapy constitutes the central pillar of treatment. Psychostimulants, such as methylphenidate and lisdexamfetamine, are considered first-line treatment, while non-stimulant drugs, such as atomoxetine, represent therapeutic alternatives. The use of these medications is associated with potential adverse reactions. This study aims to provide a critical insight into the frequency, mechanisms, and main clinical impacts associated with adverse reactions to these drugs.

**Objectives:**

Analyze the epidemiology of the main adverse reactions of methylphenidate, lisdexafetamine and atomoxetine in patients undergoing treatment for ADHD.

**Methods:**

This literature review was conducted between February 2024 and September 2025 through a systematic search of the PubMed, ScienceDirect, SciELO, and LILACS databases, as well as clinical protocols and therapeutic guidelines. Articles published in the last ten years were included, prioritizing clinical trials, systematic reviews, and meta-analyses. The keywords used were attention deficit hyperactivity disorder, ADHD, adverse reactions, medications, and side effects.

**Results:**

Methylphenidate inhibits dopamine and norepinephrine transporters, with the main adverse effects including insomnia, loss of appetite, headache, and transient increases in heart rate and blood pressure. Lisdexamfetamine increases dopamine and norepinephrine in brain regions linked to attention and behavior. Its adverse effects are similar to methylphenidate, although tachycardia and hypertension may occur early in treatment. Atomoxetine selectively inhibits norepinephrine reuptake. Adverse effects include gastrointestinal symptoms, fatigue, fatigue, and elevated blood pressure, typically self-limiting. However, it has a higher discontinuation rate than stimulants. The results were summarized in graphs highlighting adverse reactions, indicating that ADHD treatment should be based on a careful assessment of the efficacy, safety, and tolerability of each medication, considering both symptom severity and individual patient response.

**Image 1:**
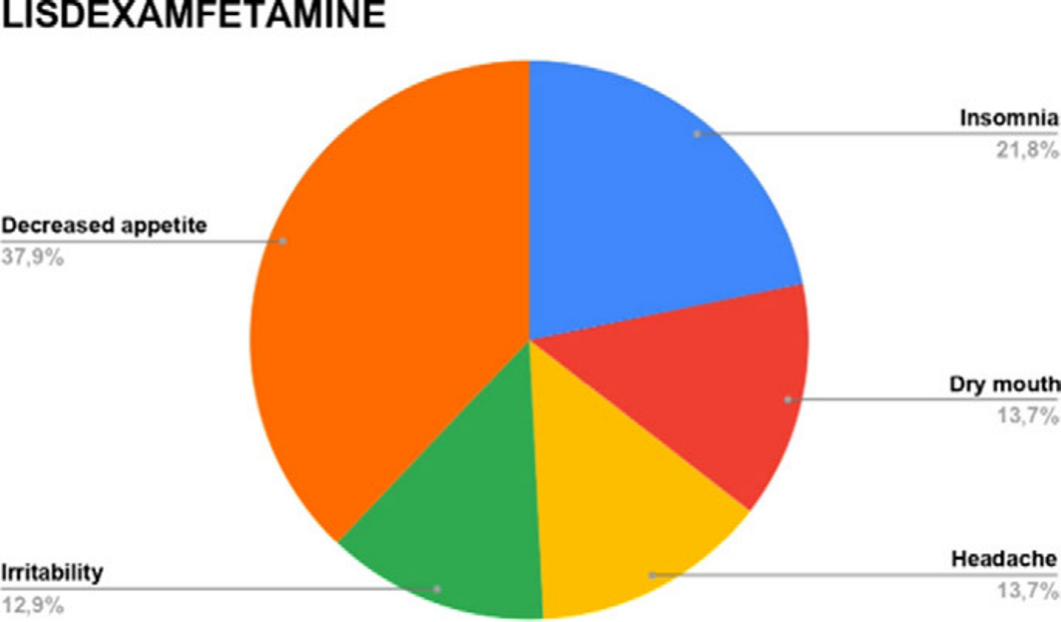

**Image 2:**
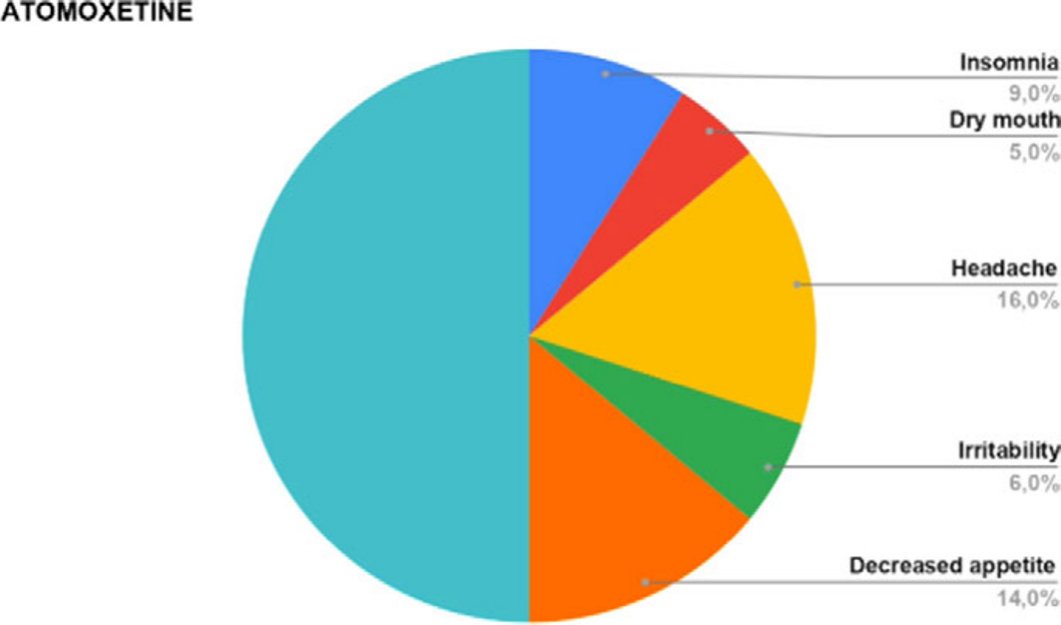

**Image 3:**
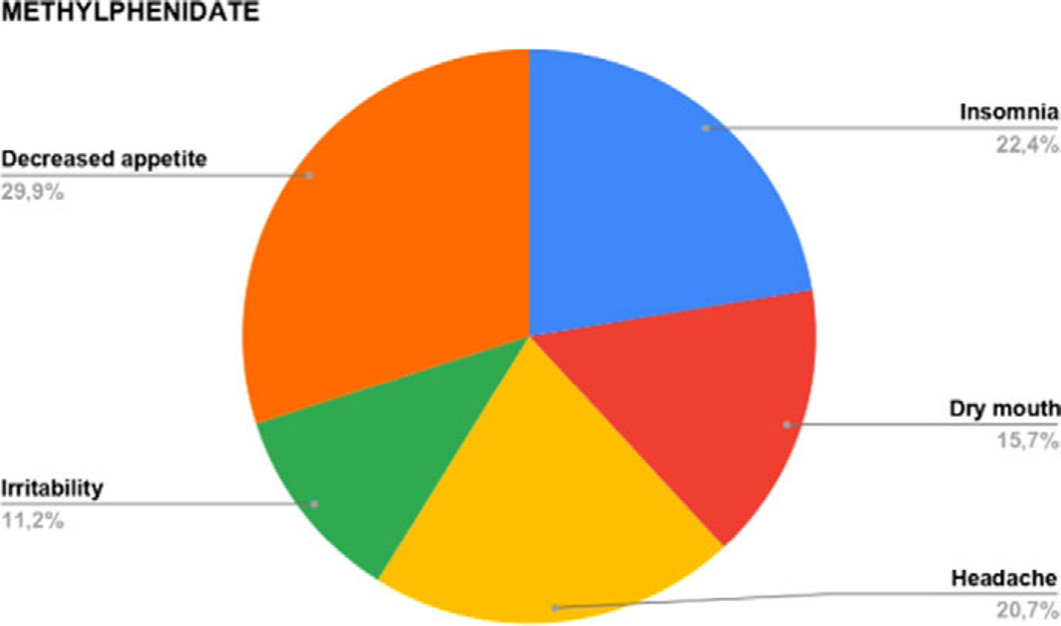

**Conclusions:**

Methylphenidate and lisdexamfetamine were linked to more frequent adverse effects, while atomoxetine showed a better safety profile but higher discontinuation rates. Psychostimulants overall had a significantly higher risk of adverse reactions than placebo. Atomoxetine differed in safety, with more gastrointestinal effects and lower tolerability in some cases. Despite many side effects decreasing over time, longer trials with representative samples are needed to better assess long-term safety.

**Disclosure of Interest:**

None Declared

